# Deficits in attentional processing of fearful facial expressions in schizophrenic patients

**DOI:** 10.1038/srep32594

**Published:** 2016-09-02

**Authors:** Yunzhe Liu, Dandan Zhang, Yanli Zhao, Shuping Tan, Yuejia Luo

**Affiliations:** 1 State Key Laboratory of Cognitive Neuroscience and Learning & IDG/McGovern Institute for Brain Research, Beijing Normal University, Beijing, China; 2 Institute of Affective and Social Neuroscience, Shenzhen University, Shenzhen, China; 3 Center for Psychiatric Research, Beijing Huilongguan Hospital, Beijing, China

## Abstract

Impaired attentional processing of negative facial expressions is prominent in schizophrenia and has been shown to be associated with patients’ social dysfunctions. However, little is known about when and which specific attention deficits influence social functions. Given the dynamic feature of attention, it is necessary to investigate the attention deficits in negative emotional processing unfolding in time. The current study used event-related potentials (ERPs) to investigate the temporal dynamics of attention deficits in emotion perception and their potential relationship with emotional/social impairments in neuroleptic naive schizophrenic patients. Two specific attention deficits were identified and were found to be associated with emotional/social impairments. More specifically, the deficit in orienting attention (evidenced with the reduced P1 amplitude) was correlated with expressive deficits, while the deficit in executive control of attention (evidenced with the reduced P3 amplitude) was correlated with avolition/asociality. Together, these findings may provide novel insights into the core pathophysiological processes and offer objective biomarkers for specific emotional/social impairments in schizophrenia. It is also hoped that this study helps to bridge the gap between basic cognitive deficits and relative high-level social dysfunctions in schizophrenic patients.

Impaired attention is often observed among schizophrenic patients and their relatives^1^. A growing body of research suggests that attention dysfunction is one of the most critical determinants of quality of life in schizophrenic patients^2^. Impaired attention is detectable even before illness onset and it tends to deteriorate when patients dealing with emotional, especially negative, information^3^,^4^. Given the important role of negative emotion in survival^5^,^6^, lack of appropriate attention to negative social stimuli would no doubt jeopardize patients’ social skills^7^,^8^. The degree of social dysfunction in schizophrenia is closely associated with the severity of negative symptoms^9^,^10^,^11^, especially those characterized by diminished expression and amotivation (i.e. avolition)^12^,^13^. However, little is known about whether attention deficits are associated with social dysfunction and relevant negative symptoms in schizophrenic patients.

Attention is a dynamic process, which comprises several functional constructs, including alerting, orienting and executive networks^14^. Numerous studies have demonstrated that the neural bases for different attention networks are largely independent^15^,^16^,^17^. Therefore, dissociation between specific domains of attention is advisable in neuroscience/clinical studies^14^,^18^. Researchers usually employ the attentional network test (ANT) to simultaneously examine the efficiency of alerting, orienting and executive attention^15^,^16^,^17^. One previous ANT study with a large sample size of schizophrenic patients has found a highly profound deficit in the executive network, a smaller but significant deficit in the orienting network and no deficit in the alerting network^19^. However, there is little knowledge about when and which specific deficits in attentional processing of negative stimuli influence social functions^20^,^21^.

Evidences in cognitive psychology have shown that orientating and executive control of attention are involved, to a large extent, sequentially in time for negative emotional processing^21^,^22^,^23^. Given the dynamic feature of attention, it would be more informative to investigate the attentional deficits in negative emotional processing unfolding in time. Understanding the relationship between the sequentially involved attention networks and emotional/social impairments is an urgent task of schizophrenic neuroscience, the answer to which would provide novel insights into the core pathophysiological processes and offer objective biomarkers for specific social impairments in schizophrenia^24^.

The event-related potential (ERP) technique was employed in this study given its high time resolution^25^. Regarding orienting network, previous ERP studies in healthy subjects have indicated that the attention orienting towards negative emotional stimuli reliably enhances the occipital P1 and the parietal P3 components, representing an early increased visual perception^26^ and a later voluntary attention allocation towards or re-orienting from negative emotions^21^, known as “negativity bias”^5^,^6^. Regarding executive control network, healthy individuals typically devote more effort to resolve cognitive conflict, which is characterized by larger P3 amplitudes. However, schizophrenic patients show difficulties in allocating attention resources when encountering conflict, accompanied by a diminished P3 component^27^. Given the acknowledged emotion deficits in patients with schizophrenia^28^,^29^, it is expected that the above mentioned ERP findings of orienting/executive function would be further deteriorated when patients process negative emotional stimuli.

Social dysfunctions in schizophrenia have received growing attention in recent years^30^,^31^. At the same time, a large body of work has explored the neural basis of basic cognitive processing in schizophrenic patients^32^. However, it is unclear whether the complicated social dysfunctions prominent in schizophrenia are related to basic cognitive deficits such as attention network. Therefore, the present study aimed to investigate the deficits in attentional processing of negative social stimuli (fearful faces here) unfolding in time, and their potential relationship with emotional/social impairments in schizophrenic patients. Studies focusing on this issue could help to bridge the gap between basic cognitive deficits and relative high-level social dysfunctions in schizophrenia^12^,^13^. Clinically, the severity of emotional/social impairments is often assessed using the Positive and Negative Syndrome Scale (PANSS)^33^. Previous studies have shown that negative rather than positive symptoms of schizophrenia consistently have two factors linked to emotional/social dysfunction^11^,^12^, which were therefore our major concerns in this study. The two factors were diminished expression (typically involving symptoms of reduced facial and vocal expressivity and reduced verbal output) and anhedonia and asociality (composed of symptoms of anhedonia, diminished interest, and decreased social engagement)^13^. We hypothesized that schizophrenic patients are characterized by sequential deficits of orienting attention and exective attention in fearful face processing. More specifically, we expect diminished amplitudes of P1 and P3 when patients orient or disengage their attentions to or from fearful faces, and reduced P3 component when patients try to inhibit task-irrelevant emotional information, compared with healthy controls. It is further expected that the ERP abnormity found in the orienting/executive attentional process of negative facial expressions would correlate with specific negative symptom factors. This study only included neuroleptic naive patients, which helped to clarify that the observed behavioral and ERP deviations in patients were free from medication influence.

## Methods

### Participants

Twenty-seven outpatients (13 females) of Beijing Huilongguan Hospital and twenty-six normal controls (12 females) in surrounding community were recruited as paid participants.

Patients were diagnosed according to the criteria for Schizophrenia in Diagnostic and Statistical Manual (DSM-IV). Patients with schizoaffective disorder, schizotypal or schizoid personality disorder were excluded. None of the patients were in a major depressive or manic episode at the time of testing. Additional exclusion criteria for patients included: 1) history of significant brain trauma, 2) neurological disorder, 3) substance abuse or dependence in the past six months, 4) mental retardation (IQ < 70), and 5) who had received electroconvulsive therapy in the past six months. At the time of experiment, all patients were untreated with medication. Indeed, they are all first episode patients, never treated with neuroleptics.

Healthy control participants were screened with the SCID^34^ and SCID-II^35^. Exclusion criteria for control participants were 1) any lifetime Axis I psychotic or mood disorders, 2) recurrent depression, 3) paranoid, schizotypal or schizoid personality disorder, 4) seizure disorder, 5) history of head injury with possible neurological sequela, 6) the presence of one or more first-degree relatives with schizophrenia, and 7) substance abuse or dependence in the past six months.

The interview and clinical symptom rating were based on consensus of two senior psychiatrists who were trained in the Center for Psychiatric Research of Beijing Huilongguan Hospital with a high reliability (κ = 0.83). There was no significant difference between the two groups with respect to age, handedness, IQ, and education (Table 1). Written informed consent was obtained prior to the experiment. The experimental protocol was approved by the Ethics Committee of Beijing Huilongguan Hospital and was in compliance with the ethical guidelines of the American Psychological Association.

### Stimuli

The cues used in the experiment were fearful and neutral faces. Facial pictures were black and white photographs selected from the native Chinese Facial Affective Picture System^36^, with equal number of facial pictures between males and females. A total of 40 faces were used (20 fearful and 20 neutral faces). Each picture had been assessed for its valence and arousal on a 9-point scale with a large sample of Chinese participants in a previous survey. Independent t-test showed that the two categories of faces have significantly different emotional valence scores (*t*(38) = −9.47, *p* < 0.001; fear = 2.68 ± 0.10, neutral = 4.27 ± 0.14) as well as arousal scores (*t*(38) = 12.3, *p* < 0.001; fear = 6.73 ± 0.23, neutral = 3.60 ± 0.11). Facial pictures (2.5° × 3°) were presented with the same contrast and brightness on the black background.

The target stimuli consisted of a row of five leftward or rightward arrows. The target arrow was surrounded by four flankers, which had either the same (congruent condition) or the opposite direction (incongruent condition). The five white arrows (3.5°) were presented to the top or bottom of a central fixation.

### Procedure

**Figure 1:**
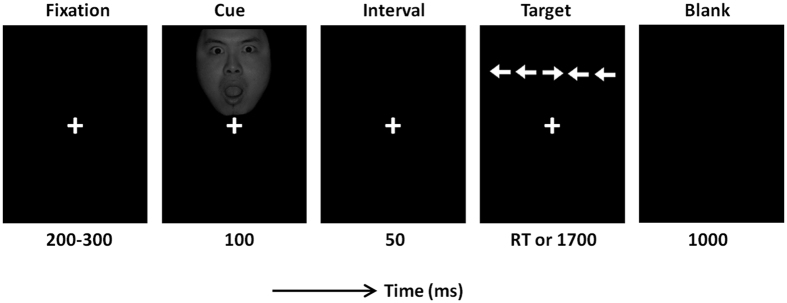
Illustration of one experimental trial in this study. RT, reaction time.

In order to assess the interactions between emotion and orienting/executive attention, the experimental procedure was modified from Fan *et al*.^17^ and Cohen *et al*.^37^. As shown in Fig. 1, each trial started with a fixation, followed by a cue that was presented to the top (50%) or the bottom (50%) of the fixation. Participants were required to respond as quickly and as accurately as possible regarding the direction of the target arrow by pressing the “left” or “right” button, respectively, on the joystick with their left or right thumb.

The experimental design was factorial, with three within-subject factors: emotion (fear *vs*. neutral), cue type (valid *vs*. invalid) and flanker type (congruent *vs*. incongruent). In valid and in invalid trials, the target stimuli appeared at the same and opposite location previously occupied by the emotional face. The total experiment consisted of 320 trials (40 trials per condition × two emotions × two cue types × two flanker types).

### EEG recording and analysis

Brain electrical activity was recorded referentially against left mastoid and off-line re-referenced to average activities over the scalp. The data were collected by a 64-channel amplifier with a sampling frequency of 250 Hz (NeuroScan Inc., Herndon, USA). Electrodes were placed on the scalp via an elastic cap according to the standard 10–20 system. Ocular artifacts were removed from EEGs using a regression procedure. The recorded EEG data were filtered (0.01–30 Hz) and segmented beginning 200 ms prior to the onset of cues and lasting for 1200 ms, followed by baseline correction and averaging. Trials contaminated with large artifacts (peak-to-peak deflection exceeded 100 μV) were excluded from the averaging. This procedure rejected 4.8 ± 0.9 trials per condition per individual (no significant difference was found between conditions and groups).

The averaged data were derived from all electrodes, but only the electrodes at which the components reached their peak values were entered into statistical analysis. Time windows for mean amplitude calculation were centered at the peak latencies of ERP components in grand-mean waveforms, with a shorter window length for early components and a longer length for late components. The mean amplitude of the P1 was calculated at the electrode sites of O1, O2, PO3, PO4 (time window = 310–360 ms after cue onset, i.e., 160–210 ms after target onset). The mean amplitude of the P3 was calculated at the electrode sites of P1, P2, Pz and CPz (time window = 450–700 ms after cue onset, i.e., 300–550 ms after target onset).

### Statistics

Descriptive data were presented as mean ± standard error. To conveniently investigate the emotion-modulation effect on orienting attention and executive attention, this study used *attention bias score* to measure the behavioral data and used *attention bias amplitude* to measure the ERP data. In particular, the attention bias scores of accuracy rate and reaction time (RT) were defined as the variable difference between validly and invalidly cued conditions, or between congruent and incongruent flanker conditions. Accordingly, two-way repeated-measures ANOVAs were performed on behavioral and ERP measurements, with emotion as the within-subject factor, and group as the between-subject factor.

According to the study of Liemburg *et al*.^38^ and Jang *et al*.^39^, the two negative symptom domains of PANSS could be calculated as follows. The Factor 1 (expressive deficits) consists of PANSS items of blunted affect (factor loading = 0.85), poor rapport (0.81), lack of spontaneity (0.83), and motor retardation (0.58). The Factor 2 (avolition/asociality) consists of PANSS items of emotional withdrawal (0.94), passive social withdrawal (0.82), and active social avoidance (0.60)^39^. In this study, two-tailed Pearson’s *r* correlation was performed between the two PANSS negative factors (Table 2) and the ERP measurements of patients. Correction for multiple comparisons was based on Holm’s stepwise method. Partial correlation was used to test correlation between negative symptoms and the ERP data while controlling for positive symptoms.

## Results

To demonstrate the independency of orienting attention and executive attention, repeated-measures ANOVAs were first performed on measurements of the accuracy rate, the RT and the P1/P3 amplitudes, with cue validity (valid vs. invalid cues) and flanker congruence (congruent vs. incongruent flankers) as within-subject factors. No significant interaction was found (the largest *F* values: *F*(1, 25) = 1.17 for the controls, *F*(1, 26) = 0.92 for the patients, and *F*(1, 52) = 1.11 for all the subjects). These results indicated the independency of the two attention networks in this study.

### Emotion effect on orienting attention

#### Behavioral data

The attention bias score of the reaction time (RT) was defined as the differential RT between invalidly and validly cued conditions.

The interaction effect of emotion by group on the attention bias score of the RT was significant (*F*(1, 51) = 8.96; *p* = 0.004; 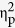 = 0.149). The attention bias score in controls (*F*(1, 25) = 6.42; *p* = 0.014) was higher in the fearful cue condition (59.2 ± 6.22 ms) compared with the neutral cue condition (44.5 ± 7.12 ms). However, the emotion effect on the attention bias score did not achieve significant level in patients (*F*(1, 26) = 2.46; *p* = 0.123).

The RT in the valid cue condition (804 ± 16.1 ms) was significantly shorter than that in the invalid cue condition (858 ± 14.9 ms; *p* < 0.001).

The results of accuracy rate in the valid cue and the invalid cue conditions were 0.953 ± 0.009 and 0.950 ± 0.009 (*p* = 0.278). No significant effect was found in the attention bias score of the accuracy rate.

#### The P1 amplitude

**Figure 2:**
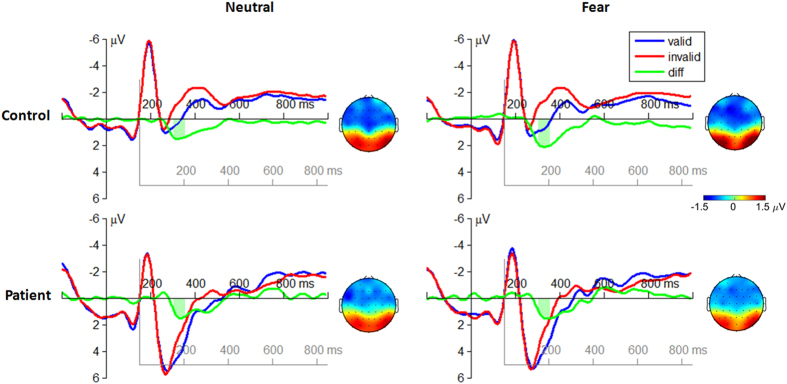
The grand-mean ERP waveforms in the valid cue and the invalid cue conditions at the electrode site of O1, O2, PO3, and PO4 (averaged data). The attention bias amplitude of the target-locked P1 component was defined as the mean differential amplitude between the valid cue and the invalid cue conditions within the time window of 160–210 ms after target onset (see the light green region in the figure). The black axes are locked to the cue; the grey axes are locked to the target. EEG topographies display the scalp distribution of the attention bias amplitudes of the P1 in different conditions.

The attention bias amplitude of the target-locked P1 component was defined as the differential amplitudes between validly and invalidly cued conditions (Fig. 2).

The interaction effect of emotion by group was significant (*F*(1, 51) = 5.22; *p* = 0.027; 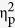 = 0.093). The attention bias amplitude of the P1 in controls (*F*(1, 25) = 11.2; *p* = 0.002) was higher in the fearful condition (2.47 ± 0.31 μV) compared with the neutral condition (1.13 ± 0.35 μV) while the emotion effect was not significant in patients (*F*(1, 26) < 1; fear = 1.61 ± 0.31 μV, neutral = 1.56 ± 0.34 μV).

The main effect of emotion was significant (*F*(1, 51) = 5.99; *p* = 0.018; 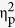 = 0.105). The attention bias amplitude in the fearful cue condition (2.04 ± 0.22 μV) was larger than that in the neutral cue condition (1.35 ± 0.24 μV).

#### The P3 amplitude

**Figure 3:**
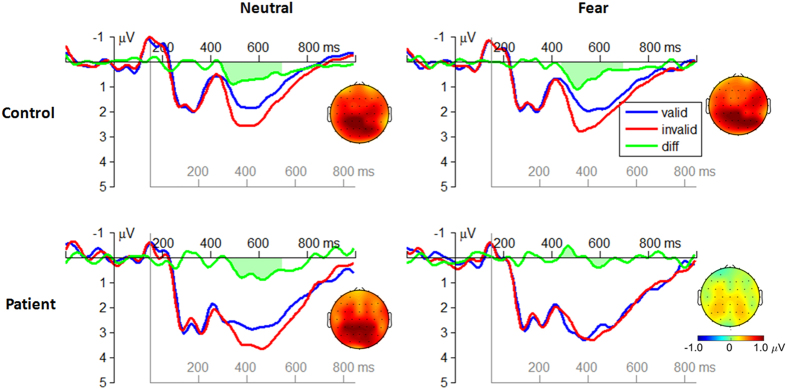
The grand-mean ERP waveforms in the valid cue and the invalid cue conditions at the electrode site of P1, P2, Pz, and CPz (averaged data). The attention bias amplitude of the target-locked P3 component was defined as the mean differential amplitude between the invalid cue and the valid cue conditions within the time window of 300–550 ms after target onset. EEG topographies display the scalp distribution of the attention bias amplitudes of the P3 in different conditions.

The attention bias amplitude of the target-locked P3 component was defined as the differential amplitude between invalidly and validly cued condition (Fig. 3).

The interaction effect of emotion by group was significant (*F*(1, 51) = 6.66; *p* = 0.013; 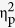 = 0.115). The attention bias amplitude of the P3 in patients (*F*(1, 26) = 12.9; *p* = 0.001) was smaller in the fearful cue condition (0.01 ± 0.18 μV) compared with the neutral cue condition (0.65 ± 0.15 μV) while the emotion effect was not significant in controls (*F*(1, 25) <1; fear = 0.65 ± 0.16 μV, neutral = 0.68 ± 0.18 μV).

The main effect of group was significant (*F*(1, 51) = 5.76; *p* = 0.020; 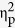 = 0.101). The attention bias amplitude evoked in the patients (0.34 ± 0.13 μV) was smaller than that evoked in the controls (0.65 ± 0.11 μV).

#### Correlations

Correlations were performed between the two negative symptom factors of PANSS and the attention bias amplitudes of the P1 and the P3 in fearful- and neutral-cued conditions. Totally 8 correlations (2 × 2 × 2) were performed in this section.

**Figure 4:**
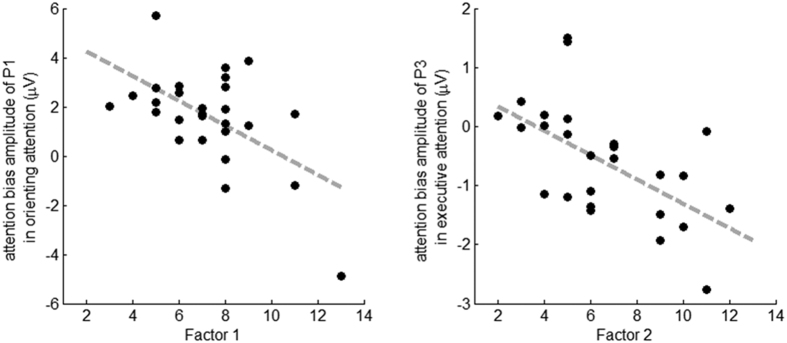
Correlations between the two negative symptom factors of PANSS and the attention bias amplitudes of the P1 and the P3 in orienting and executive attention networks. The x-axis is the factor score of the patients and the y-axis is the ERP amplitude. Factor 1-expressive deficits, Factor 2-avolition/asociality.

Results showed only one significant correlation after correction for multiple comparisons. The Factor 1 (expressive deficits) correlated significantly with the attention bias amplitude of the P1 in the fearful cue condition (*r* = −0.57, *p* = 0.002, corrected *p* = 0.016; Fig. 4). After controlling for the score of Positive scale in the PANSS, the partial correlation coefficients between Factor 1 and the attention bias amplitude of the P1 did not change greatly (*r* = −0.54, *p* = 0.005).

### Emotion effect on executive attention

#### Behavioral data

The attention bias score of the RT was defined as the differential RT between the incongruent flanker condition and the congruent flanker conditions.

The interaction effect of emotion by group on the attention bias score of the RT was significant (*F*(1, 51) = 5.14; *p* = 0.028; 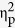 = 0.092). The attention bias score in controls (*F*(1, 25) = 8.41; *p* = 0.005) was higher in the fearful cue condition (31.4 ± 4.70 ms) compared with the neutral cue condition (16.4 ± 4.88 ms). However, the emotion effect on the attention bias score did not achieve significant level in patients (*F*(1, 26) < 1).

The RT in the congruent flanker condition (820 ± 14.9 ms) was significantly shorter than that in the incongruent flanker condition (841 ± 16.0 ms; *p* < 0.001).

The accuracy rate in the congruent flanker condition (0.957 ± 0.009) was significantly higher than that in the incongruent flanker condition (0.946 ± 0.010; *p* < 0.001). No significant effect was found in the attention bias score of the accuracy rate.

#### The P1 amplitude

The attention bias amplitude of the target-locked P1 component was defined as the differential amplitude between the incongruent flanker condition and the congruent flanker condition. No significant effect was found in the data.

#### The P3 amplitude

**Figure 5:**
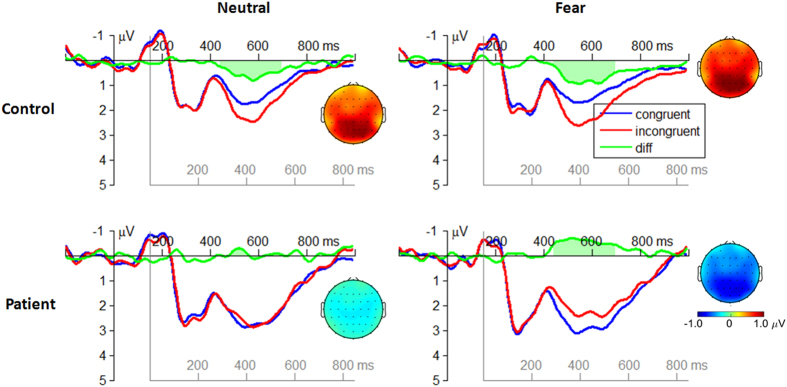
The grand-mean ERP waveforms in the congruent flanker and the incongruent flanker conditions at the electrode site of P1, P2, Pz, and CPz (averaged data). The attention bias amplitude of the target-locked P3 component was defined as the mean differential amplitude between the incongruent flanker and the congruent flanker conditions within the time window of 300–550 ms after target onset. EEG topographies display the scalp distribution of the attention bias amplitudes of the P3 in different conditions.

The attention bias amplitude of the target-locked P3 component was defined as the differential amplitude between the incongruent flanker condition and the congruent flanker condition (Fig. 5).

The interaction effect of emotion by group was significant (*F*(1, 51) = 7.57; *p* = 0.008; 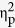 = 0.129). The attention bias amplitude of the P3 in patients (*F*(1, 26) = 7.38; *p* = 0.009) was smaller in the fearful cue condition (−0.60 ± 0.18 μV) compared with the neutral cue condition (−0.10 ± 0.17 μV) while the emotion effect was not significant in controls (*F*(1, 25) = 1.24, *p* = 0.271; fear = 0.73 ± 0.18 μV, neutral = 0.51 ± 0.17 μV).

The main effect of group was significant (*F*(1, 51) = 21.3; *p* < 0.001; 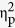 = 0.294). The attention bias amplitude evoked in the patients (−0.35 ± 0.15 μV) was smaller than that evoked in the controls (0.62 ± 0.15 μV).

#### Correlations

Correlations were performed between the two negative symptom factors of PANSS and the attention bias amplitudes of the P3 in fearful- and neutral-cued conditions (totally 2 × 2 = 4 correlations).

Results showed only one significant correlation after correction for multiple comparisons. The Factor 2 (avolition/asociality, *r* = −0.58; *p* = 0.002; corrected *p* = 0.006) correlated significantly with the attention bias amplitude of the P3 in the fearful cue condition (Fig. 4). After controlling for the score of Positive scale in the PANSS, the partial correlation coefficients between Factor 2 and the attention bias amplitude of the P3 did not change greatly (*r* = −0.57, *p* = 0.002).

## Discussion

The present results identified two separate mechanisms that link specific attention deficits to different emotional/social impairments in schizophrenia. While deficits in orienting attention were correlated with the first factor of negative symptoms in patients (expressive deficits), deficits in executive control of attention were associated with the second factor of negative symptoms (avolition/asociality).

In the orienting attention network, our result revealed that schizophrenic patients were able to allocate their attention towards valid cues, evidenced with the enhanced amplitudes of the target-locked P1 component. However, the attention bias to negative stimuli was not observed in the patients, who showed similar attention bias amplitudes of the P1 between neutral and fearful conditions (Fig. 2), and even attenuated attention bias amplitudes of the P3 in fearful condition compared with neutral condition (Fig. 3). The diminished orienting attention to negative facial expressions (i.e., disappearance of “negativity bias”) in schizophrenia is well in line with previous studies^40^. For instance, schizophrenic patients were found to have attenuated P1 amplitudes compared with normal subjects when they attended to negative emotional stimuli^20^; and patients displayed reduced amygdala activity, compared with controls, when they perceived fearful faces in both conscious and nonconscious conditions^41^. The “negativity bias” refers to a general tendency to process negative emotional information with priority to positive and neutral information^5^. It has been demonstrated that such quick response mainly depends on a visual pathway preferentially tuned to coarse-magnocellular inputs (i.e. low spatial-frequency information^42^,^43^). Our result suggests that the lack of “negativity bias” in involuntary orienting may be due to a bottom-up dysfunction within the early visual pathway in schizophrenia^40^,^44^. While the P1 serves as a biomarker of involuntary attention orientation, the later P3 may function as an indicator of voluntary regulation of attention^21^,^45^. More specifically, when the cue is invalid, participants had to re-orientate their attention to the location of the target in the dot-probe task. According to the principle of least effort^46^,^47^, this procedure would require more cognitive resources and a larger effort of top-down regulation compared to the attention processing in the validly cued condition. Therefore the increased P3 amplitude in the invalid condition may reflect the degree of effort involved in the top-down regulation of attention, as also suggested by previous studies^21^,^45^,^48^,^49^,^50^. The result in Fig. 3 showed that the P3 displayed higher amplitudes in invalid condition compared with valid condition in the controls^21^; however, this pattern did not exist for the fearful cued condition in the patients. This result indicates that patients cannot appropriate re-orient his/her attention from negative emotion to goal-related direction.

More interestingly, the diminished orienting attention towards fearful faces (indexed by the attention bias amplitude of the P1) was significantly correlated with Factor 1 of the negative symptoms (i.e., expressive deficits) in schizophrenic patients (Fig. 4). Expressive deficits, which means reduced emotional expressions in social interactions, has been proved to be associated with impaired performances of affect perception tasks (e.g. facial expression recognition and discrimination)^51^,^52^. In addition, it has been reported that the severity of blunted affect can independently predict the performance of emotional intensity differentiation^53^. By disassociating attention constructs in the current study, it is suggested that the diminished expression in the patients is specifically associated with a failure in automatic orienting to salient signals. Though the exact causal mechanism remains unclear, one possible explanation might be that a failure in automatic orienting to salient signals may lead to patients’ failure in identifying facial expressions of others^20^. Patients are thus less likely to express their own feelings, resulting in diminished expression^54^.

In the executive attention network, schizophrenic patients failed to recruit enough attentional resources to resolve the flanker conflict, evidenced by the diminished amplitudes of the target-locked P3 component in incongruent condition compared to congruent one. Furthermore, such deficit became even worse when the patients were interfered by negative emotion (Fig. 5). The impaired ability to inhibit emotional information allows negative affective stimuli to exert inappropriate influence on conflict-resolving function. This result is consistent with previous studies indicating that schizophrenic patients have an impaired ability to regulate the influence of irrelevant negative affective information on cognitive processes^55^,^56^. Furthermore, the deficits in executive attention may be specific to social-related information, since it has been reported that patients showed no inhibitory deficit in the Stroop task when social-unrelated negative words were used^57^, while they exhibited impairments of executive control when dealing with social emotional information (e.g. negative emotional faces)^18^,^58^. Both frontal and limbic dysfunctions (e.g. the hypo-activation of anterior cingulate cortex^59^ and dorsolateral prefrontal cortex (DLPFC)^60^,^61^) are responsible for the deficit of executive control of attention in schizophrenia^62^. This deficit may in turn prevent the brain from inhibiting irrelevant emotional information and disturb goal-directed behaviors^63^,^64^.

In addition, it is found that patients’ abolished executive control of attention to fearful faces (indexed by the attention bias amplitude of the P3) was significantly correlated with the severity of avolition/asociality (Fig. 4). Asociality is defined as a state with diminished inspiration to participate in social activities. It is proposed that without appropriate executive control of attention to negative emotion, schizophrenic patients are unable to inhibit negative affective information when it is irrelevant to the goal-directed behavior, thus resulting in a withdrawal of any unnecessary unpleasant experiences^58^, which in turn makes patients have little interest in socializing^3^. Social skill training, such as an explicit instruction on how to deal with negative social information, is likely to improve such social impairments in schizophrenic patients^65^,^66^. Psychopharmacological approaches are also used for effective relief of the amotivation-induced social impairments. For example, after taking apomorphine (a non-selective dopamine agonist which activates both D1-like and D2-like receptors), schizophrenic patients display enhanced activation of anterior cingulate cortex and improved willing to participate in social interaction^67^,^68^.

It was also interesting to note that in the current study, the P3 amplitude was overall larger in the patients compared with the controls (Figs 2 and 5), which was distinct to most previous findings^27^,^69^,^70^. Actually, both hyper- and hypo-activity of ERP-measured P3 component and BOLD-measured prefrontal cortex have been identified in schizophrenic patients^71^. For instance, it was found in working memory task that patients have larger P3 amplitudes^72^ and enhanced DLPFC activity^73^ along with poorer behavioral performances, compared with healthy controls. A recent meta-analysis^71^ further revealed that the DLPFC inefficiency might be manifested in either direction depending on task demands; when cognitive load is minimal to moderate (which is likely to be the case in our study), the DLPFC engagement is greater in the schizophrenic subjects than in controls. However, it is currently unknown whether the observed inefficiency in attention control is a general deficit or more specific to emotion perception.

Finally, readers may notice an atypical use of the component term in the present analysis. The typical peak latency for the P1component is 100–130 ms (Luck, 2005). However, we used the term P1 to name the relatively blunt component (rather than a sharp one) with the peak latency of approximately 185 ms post target onset (Fig. 2). We made this designation mainly because the scalp topography of this component was consistent with the expectation for lateral occipital P1 (Luck, 2005). The latency delay observed for the P1 component is likely attributable to its calculation method, i.e., this component was examined based on the differential waveforms between valid and invalid conditions (the P1 peaked earlier in the original ERP waveforms before waveform subtraction).

In summary, we find two separate mechanisms that link specific attention deficits to different emotional/social impairments. While deficits in orienting attention correlate with diminished expression, deficits in executive control of attention are found to be associated with avolition/asociality. Moreover, the inclusion of neuroleptic naive patients helped clarify the behavioral and ERP deviations found in this study. Though the exact causal mechanism remains unclear, we propose that the failure in orienting to salient social information may be responsible for the diminished expression in patients. The deficits in executive control of attention prevent patients to appropriately resolve social conflicts, resulting in avolition and less socializing. Together, these findings may shed some light on resolving the heterogeneity of schizophrenia, particularly with respect to the variety of impairments in social functions found in this disorder.

## Additional Information

**How to cite this article**: Liu, Y. *et al*. Deficits in attentional processing of fearful facial expressions in schizophrenic patients. *Sci. Rep.* **6**, 32594; doi: 10.1038/srep32594 (2016).

## Figures and Tables

**Table 1:** Demographic and clinical data of patients and control groups.

Characteristics	Patient (n = 27)	Control (n = 26)	Statistics
Mean age, y	21.6 (18–26)	23.2 (17–27)	*t* (51) = −1.64, *p* = 0.107
Education time, y	13.3 (9–16)	14.2 (9–16)	*t* (51) = 1.42, *p* = 0.161
Handedness, right/left	27/0	26/0	
IQ^a^	101 (78–123)	103 (79–125)	*t* (51) = −0.68, *p* = 0.498
PANSS score^b^			
Positive scale	14.4 (12–34)	9.2 (7–17)	*p* < 0.001
Negative scale	16.2 (10–30)	10.4 (8–20)	*p* < 0.001
General Psychopathology scale	31.5 (26–50)	27.4 (25–31)	*p* < 0.001

Descriptive data are presented as mean (range) or mean ± standard deviation.

^a^The Wechsler Adult Intelligence Scale (fourth edition)^74^,^75^,^76^.

^b^Positive and Negative Syndrome Scale^33^.

**Table 2:** The two negative symptom factors of PANSS. The factor loadings were based on the study of Jang *et al*.^39^.

Subject number	Factor 1	Score of PANSS items and factor loadings				Factor 2	Score of PANSS items and factor loadings		
		N1 0.85	N3 0.81	N6 0.83	G7 0.58		N2 0.94	N4 0.82	G16 0.60
1	9	3	3	3	3	6	3	2	2
2	5	2	1	1	3	6	3	2	3
3	7	3	2	1	3	5	1	3	3
4	6	1	3	1	3	4	3	1	1
5	7	3	2	2	2	7	3	4	2
6	8	3	3	2	2	6	3	3	1
7	5	2	1	1	3	6	3	3	1
8	7	3	3	1	2	7	3	3	3
9	6	3	3	1	1	11	5	4	5
10	6	3	3	1	1	9	4	3	4
11	8	3	3	1	3	6	3	2	2
12	11	5	5	1	3	12	5	5	5
13	4	1	1	1	3	5	2	2	2
14	3	1	1	1	1	3	2	1	1
15	9	3	3	3	3	4	2	2	1
16	8	2	3	2	3	5	1	3	2
17	6	3	2	1	2	3	1	2	1
18	11	5	4	2	3	7	3	3	3
19	8	4	3	1	2	10	4	5	4
20	13	5	5	4	2	11	5	5	4
21	8	4	3	1	2	9	4	4	3
22	8	4	3	1	2	10	5	4	3
23	7	4	3	1	1	9	4	4	4
24	5	3	1	1	1	5	2	3	1
25	5	2	1	2	1	4	1	1	3
26	8	3	2	3	3	2	1	1	1
27	8	3	2	2	3	5	3	2	1

Factor 1- expressive deficits, Factor 2-avolition/asociality.

N1-blunted affect, N2-emotional withdrawal, N3-poor rapport, N4-passive/apathetic social withdrawal, N6-lack of spontaneity and flow of conversation, G7-motor retardation, G16-active social avoidance.

## References

[b1] Cornblatt B. A. & Keilp J. G. Impaired attention, genetics, and the pathophysiology of schizophrenia. Schizophr Bull 20, 31–46 (1994). 8197420 10.1093/schbul/20.1.31

[b2] Nuechterlein K. H. . Neurocognitive predictors of work outcome in recent-onset schizophrenia. Schizophr Bull 37, S33–S40 (2011). 21860045 10.1093/schbul/sbr084 PMC3160123

[b3] Williams L. M. . Dysregulation of arousal and amygdala-prefrontal systems in paranoid schizophrenia. Am J Psychiat 161, 480–489 (2004). 14992974 10.1176/appi.ajp.161.3.480

[b4] Kohler C. G., Bilker W., Hagendoorn M., Gur R. E. & Gur R. C. Emotion recognition deficit in schizophrenia: association with symptomatology and cognition. Biol Psychiatry 48, 127–136 (2000). 10903409 10.1016/s0006-3223(00)00847-7

[b5] Rozin P. & Royzman E. B. Negativity bias, negativity dominance, and contagion. Pers Soc Psychol Rew 5, 296–320 (2001).

[b6] Vaish A., Grossmann T. & Woodward A. Not all emotions are created equal: the negativity bias in social-emotional development. Psychol Bull 134, 383–403 (2008). 18444702 10.1037/0033-2909.134.3.383 PMC3652533

[b7] Hooker C. & Park S. Emotion processing and its relationship to social functioning in schizophrenia patients. Psychiatry Res 112, 41–50 (2002). 12379449 10.1016/s0165-1781(02)00177-4

[b8] Kee K. S., Green M. F., Mintz J. & Brekke J. S. Is emotion processing a predictor of functional outcome in schizophrenia? Schizophr Bull 29, 487–497 (2003). 14609242 10.1093/oxfordjournals.schbul.a007021

[b9] Blanchard J. J. & Cohen A. S. The structure of negative symptoms within schizophrenia: implications for assessment. Schizophr Bull 32, 238–245 (2006). 16254064 10.1093/schbul/sbj013 PMC2632211

[b10] Möller H.-J. Clinical evaluation of negative symptoms in schizophrenia. Eur Psychiatry 22, 380–386 (2007). 17524626 10.1016/j.eurpsy.2007.03.010

[b11] Foussias G. & Remington G. Negative symptoms in schizophrenia: avolition and Occam’s razor. Schizophr Bull 36, 359–369 (2010). 18644851 10.1093/schbul/sbn094 PMC2833114

[b12] Messinger J. W. . Avolition and expressive deficits capture negative symptom phenomenology: implications for DSM-5 and schizophrenia research. Clin Psychol Rev 31, 161–168 (2011). 20889248 10.1016/j.cpr.2010.09.002 PMC2997909

[b13] Blanchard J. J. & Cohen A. S. The structure of negative symptoms within schizophrenia: implications for assessment. Schizophr Bull 32, 238–245 (2006). 16254064 10.1093/schbul/sbj013 PMC2632211

[b14] Posner M. I. Imaging attention networks. Neuroimage 61, 450–456 (2012). 22227132 10.1016/j.neuroimage.2011.12.040 PMC3345293

[b15] Posner M. I., Sheese B. E., Odludaş Y. & Tang Y. Analyzing and shaping human attentional networks. Neural Netw 19, 1422–1429 (2006). 17059879 10.1016/j.neunet.2006.08.004

[b16] Fan J., McCandliss B. D., Fossella J., Flombaum J. I. & Posner M. I. The activation of attentional networks. Neuroimage 26, 471–479 (2005). 15907304 10.1016/j.neuroimage.2005.02.004

[b17] Fan J., McCandliss B. D., Sommer T., Raz A. & Posner M. I. Testing the efficiency and independence of attentional networks. J Cogn Neurosci 14, 340–347 (2002). 11970796 10.1162/089892902317361886

[b18] Tully L. M., Lincoln S. H. & Hooker C. I. Impaired executive control of emotional information in social anhedonia. Psychiatry Res 197, 29–35 (2012). 22425470 10.1016/j.psychres.2011.12.023

[b19] Wang K., Fan J., Dong Y., Wang C.-Q., Lee T. & Posner M. I. Selective impairment of attentional networks of orienting and executive control in schizophrenia. Schizophr Res 78, 235–241 (2005). 16154056 10.1016/j.schres.2005.01.019

[b20] De Sanctis P. . Early sensory-perceptual processing deficits for affectively valenced inputs are more pronounced in schizophrenia patients with a history of violence than in their non-violent peers. Soc Cogn Affect Neurosci 8, 678–687 (2013). 22563006 10.1093/scan/nss052 PMC3739916

[b21] Liu Y., Zhang D. & Luo Y. How disgust facilitates avoidance: an ERP study on attention modulation by threats. Soc Cogn Affect Neurosci 10, 598–604 (2015). 24974395 10.1093/scan/nsu094 PMC4381247

[b22] Carretié L., Martín-Loeches M., Hinojosa J. A. & Mercado F. Emotion and attention interaction studied through event-related potentials. J Cogn Neurosci 13, 1109–1128 (2001). 11784449 10.1162/089892901753294400

[b23] Compton R. J. The interface between emotion and attention: A review of evidence from psychology and neuroscience. Behav Cogn Neurosci Rev 2, 115–129 (2003). 13678519 10.1177/1534582303255278

[b24] Penn D. L., Sanna L. J. & Roberts D. L. Social cognition in schizophrenia: an overview. Schizophr Bull 34, 408–411 (2008). 18375928 10.1093/schbul/sbn014 PMC2632430

[b25] Luck S. J. An introduction to the event-related potential technique : MIT press, (2014).

[b26] Pourtois G., Grandjean D., Sander D. & Vuilleumier P. Electrophysiological correlates of rapid spatial orienting towards fearful faces. Cereb Cortex 14, 619–633 (2004). 15054077 10.1093/cercor/bhh023

[b27] Weisbrod M., Kiefer M., Marzinzik F. & Spitzer M. Executive control is disturbed in schizophrenia: evidence from event-related potentials in a Go/NoGo task. Biol Psychiatry 47, 51–60 (2000). 10650449 10.1016/s0006-3223(99)00218-8

[b28] Kring A. M. & Moran E. K. Emotional response deficits in schizophrenia: insights from affective science. Schizophr Bull 34, 819–834 (2008). 18579556 10.1093/schbul/sbn071 PMC2632476

[b29] Sachs G., Steger-Wuchse D., Kryspin-Exner I., Gur R. C. & Katschnig H. Facial recognition deficits and cognition in schizophrenia. Schizophr Res 68, 27–35 (2004). 15037337 10.1016/S0920-9964(03)00131-2

[b30] Brunet-Gouet E. & Decety J. Social brain dysfunctions in schizophrenia: a review of neuroimaging studies. Psychiatry Res 148, 75–92 (2006). 17088049 10.1016/j.pscychresns.2006.05.001

[b31] Pinkham A. E. Social cognition in schizophrenia. J Clin Psychiatry 75, 14–19 (2014). 24919166 10.4088/JCP.13065su1.04

[b32] Cannon T. D. How schizophrenia develops: cognitive and brain mechanisms underlying onset of psychosis. Trends Cogn Sci 19, 744–756 (2015). 26493362 10.1016/j.tics.2015.09.009 PMC4673025

[b33] Kay S. R., Flszbein A. & Opfer L. A. The positive and negative syndrome scale (PANSS) for schizophrenia. Schizophr Bull 13, 261–276 (1987). 3616518 10.1093/schbul/13.2.261

[b34] First M., Spitzer R., Gibbon M. & Williams J. Structured Clinical Interview for DSM-IV-TR Axis I Disorders, Research Version, Non-Patient Edition (SCID-I/NP). New York, New York State Psychiatric Institute (2002).

[b35] Maffei C. . Interrater reliability and internal consistency of the structured clinical interview for DSM-IV axis II personality disorders (SCID-II), version 2.0. J Pers Disord 11, 279–284 (1997). 9348491 10.1521/pedi.1997.11.3.279

[b36] Gong X., Huang Y., Wang Y. & Luo Y. Revision of the Chinese facial affective picture system. Chinese Mental Health Journal 25, 40–46 (2011).

[b37] Cohen N., Henik A. & Mor N. Can emotion modulate attention? Evidence for reciprocal links in the Attentional Network Test. Exp Psychol 58, 171–179 (2011). 20705545 10.1027/1618-3169/a000083

[b38] Liemburg E. . Two subdomains of negative symptoms in psychotic disorders: established and confirmed in two large cohorts. J Psychiatr Res 47, 718–725 (2013). 23472837 10.1016/j.jpsychires.2013.01.024

[b39] Jang S. K. . A two-factor model better explains heterogeneity in negative symptoms: evidence from the Positive and Negative Syndrome Scale. Front Psychol 7, 707 (2016). 27242619 10.3389/fpsyg.2016.00707 PMC4863882

[b40] Seok J. H. . Behavioural evidence of blunted and inappropriate affective responses in schizophrenia: lack of a ‘negativity bias’. Psychiatry Res 142, 53–66 (2006). 16730072 10.1016/j.psychres.2005.08.021

[b41] Das P. . Functional disconnections in the direct and indirect amygdala pathways for fear processing in schizophrenia. Schizophr Res 90, 284–294 (2007). 17222539 10.1016/j.schres.2006.11.023

[b42] De Cesarei A. & Codispoti M. Spatial frequencies and emotional perception. Rev Neurosci 24, 89–104 (2013). 23183741 10.1515/revneuro-2012-0053

[b43] De Cesarei A., Mastria S. & Codispoti M. Early spatial frequency processing of natural images: an ERP study. PLoS One 8, e65103 (2013). 23741468 10.1371/journal.pone.0065103 PMC3669057

[b44] Butler P. D. . Subcortical visual dysfunction in schizophrenia drives secondary cortical impairments. Brain 130, 417–430 (2007). 16984902 10.1093/brain/awl233 PMC2072909

[b45] Pollak S. D. & Tolley-Schell S. A. Selective attention to facial emotion in physically abused children. J Abnorm Psychol 112, 323–335 (2003). 12943012 10.1037/0021-843x.112.3.323

[b46] Zipf G. K. *Human Behavior and the Principle of Least Effort: An Introduction to Human Ecology*. Ravenio Books (2016).

[b47] Kurzban R., Duckworth A., Kable J. W. & Myers J. An opportunity cost model of subjective effort and task performance. Behav Brain Sci 36, 661–679 (2013). 24304775 10.1017/S0140525X12003196 PMC3856320

[b48] Sass S. M., Evans T. C., Xiong K., Mirghassemi F. & Tran H. Attention training to pleasant stimuli in anxiety. Biol Psychol 10.1016/j.biopsycho.2016.03.003 (2016). 26969581

[b49] Leutgeb V., Sarlo M., Schöngassner F. & Schienle A. Out of sight, but still in mind: electrocortical correlates of attentional capture in spider phobia as revealed by a ‘dot probe’ paradigm. Brain Cogn 93, 26–34 (2015). 25500187 10.1016/j.bandc.2014.11.005

[b50] Kappenman E. S., MacNamara A. & Proudfit G. H. Electrocortical evidence for rapid allocation of attention to threat in the dot-probe task. Soc Cogn Affect Neurosci 10, 577–583 (2015). 25062842 10.1093/scan/nsu098 PMC4381248

[b51] Shaw R. J., Dong M., Lim K. O., Faustman W. O., Pouget E. R. & Alpert M. The relationship between affect expression and affect recognition in schizophrenia. Schizophr Res 37, 245–250 (1999). 10403196 10.1016/s0920-9964(98)00172-8

[b52] Heimberg C., Gur R. E., Erwin R. J., Shtasel D. L. & Gur R. C. Facial emotion discrimination: III. Behavioral findings in schizophrenia. Psychiatry Res 42, 253–265 (1992). 1496057 10.1016/0165-1781(92)90117-l

[b53] Gur R. E. . Flat affect in schizophrenia: relation to emotion processing and neurocognitive measures. Schizophr Bull 32, 279–287 (2006). 16452608 10.1093/schbul/sbj041 PMC2632232

[b54] Williams L. M., Loughland C. M., Gordon E. & Davidson D. Visual scanpaths in schizophrenia: is there a deficit in face recognition? Schizophr Res 40, 189–199 (1999). 10638857 10.1016/s0920-9964(99)00056-0

[b55] Strauss G. P., Kappenman E. S., Culbreth A. J., Catalano L. T., Lee B. G. & Gold J. M. Emotion regulation abnormalities in schizophrenia: cognitive change strategies fail to decrease the neural response to unpleasant stimuli. Schizophr Bull 39, 872–883 (2013). 23314192 10.1093/schbul/sbs186 PMC3686456

[b56] Horan W. P., Hajcak G., Wynn J. K. & Green M. F. Impaired emotion regulation in schizophrenia: evidence from event-related potentials. Psychol Med 43, 2377–2391 (2013). 23360592 10.1017/S0033291713000019 PMC3963439

[b57] Mohanty A., Heller W., Koven N. S., Fisher J. E., Herrington J. D. & Miller G. A. Specificity of emotion-related effects on attentional processing in schizotypy. Schizophr Res 103, 129–137 (2008). 18440784 10.1016/j.schres.2008.03.003 PMC2881633

[b58] Suslow T., Roestel C. & Arolt V. Affective priming in schizophrenia with and without affective negative symptoms. Eur Arch Psychiatry Clin Neurosci 253, 292–300 (2003). 14714118 10.1007/s00406-003-0443-4

[b59] Minzenberg M. J., Laird A. R., Thelen S., Carter C. S. & Glahn D. C. Meta-analysis of 41 functional neuroimaging studies of executive function in schizophrenia. Arch Gen Psychiatry 66, 811–822 (2009). 19652121 10.1001/archgenpsychiatry.2009.91 PMC2888482

[b60] Braver T. S., Barch D. M. & Cohen J. D. Cognition and control in schizophrenia: a computational model of dopamine and prefrontal function. Biol Psychiatry 46, 312–328 (1999). 10435197 10.1016/s0006-3223(99)00116-x

[b61] Morey R. A., Inan S., Mitchell T. V., Perkins D. O., Lieberman J. A. & Belger A. Imaging frontostriatal function in ultra-high-risk, early, and chronic schizophrenia during executive processing. Arch Gen Psychiatry 62, 254–262 (2005). 15753238 10.1001/archpsyc.62.3.254 PMC2732718

[b62] Barch D. M., Sheline Y. I., Csernansky J. G. & Snyder A. Z. Working memory and prefrontal cortex dysfunction: specificity to schizophrenia compared with major depression. Biol Psychiatry 53, 376–384 (2003). 12614990 10.1016/s0006-3223(02)01674-8

[b63] Ochsner K. N. & Gross J. J. Cognitive emotion regulation insights from social cognitive and affective neuroscience. Curr Dir Psychol Sci 17, 153–158 (2008). 25425765 10.1111/j.1467-8721.2008.00566.x PMC4241349

[b64] Do K. Q., Cabungcal J. H., Frank A., Steullet P. & Cuenod M. Redox dysregulation, neurodevelopment, and schizophrenia. Curr Opin Neurobiol 19, 220–230 (2009). 19481443 10.1016/j.conb.2009.05.001

[b65] Horan W. P., Kern R. S., Green M. F. & Penn D. L. Social cognition training for individuals with schizophrenia: emerging evidence. Am J Psychiatr Rehabil 11, 205–252 (2008).

[b66] Pilling S. . Psychological treatments in schizophrenia: I. Meta-analysis of family intervention and cognitive behaviour therapy. Psychol Med 32, 763–782 (2002). 12171372 10.1017/s0033291702005895

[b67] Dolan R. J., Fletcher P., Frith C. D., Friston K. J., Frackowiak R. S. J. & Grasby P. M. Dopaminergic modulation of impaired cognitive activation in the anterior cingulate cortex in schizophrenia. Nature 378, 180–182 (1995). 7477319 10.1038/378180a0

[b68] Wise R. A. Dopamine and reward: the anhedonia hypothesis 30 years on. Neurotox Res 14, 169–183 (2008). 19073424 10.1007/BF03033808 PMC3155128

[b69] Jeon Y. W. & Polich J. Meta-analysis of P300 and schizophrenia: patients, paradigms, and practical implications. Psychophysiology 40, 684–701 (2003). 14696723 10.1111/1469-8986.00070

[b70] Picton T. W. The P300 wave of the human event-related potential. J Clin Neurophysiol 9, 456–479 (1992). 1464675 10.1097/00004691-199210000-00002

[b71] Potkin S. . Working memory and DLPFC inefficiency in schizophrenia: the FBIRN study. Schizophr Bull 35, 19–31 (2009). 19042912 10.1093/schbul/sbn162 PMC2643959

[b72] Zhao Y. L. . Dysfunction in different phases of working memory in schizophrenia: evidence from ERP recordings. Schizophr Res 133, 112–119 (2011). 22014837 10.1016/j.schres.2011.09.017

[b73] Manoach D. S. . Schizophrenic subjects activate dorsolateral prefrontal cortex during a working memory task, as measured by fMRI. Biol Psychiatry 45, 1128–1137 (1999). 10331104 10.1016/s0006-3223(98)00318-7

[b74] Wechsler D. Wechsler Adult Intelligence Scale (4th ed.) *Administration and Scoring Manual*. San Antonio: Psychological Corporation (2008).

[b75] Wechsler D. Wechsler Adult Intelligence Scale (4th ed.) *Technical and Interpretive Manual*. San Antonio: Psychological Corporation (2008).

[b76] Wang J. . Reliability and construct validity of Chinese version of Wechsler Adult Intelligence Scale-Fourth Edition. Chinese Mental Health Journal 27, 62–67 (2013).

